# Possible role of L-form switching in recurrent urinary tract infection

**DOI:** 10.1038/s41467-019-12359-3

**Published:** 2019-09-26

**Authors:** Katarzyna M. Mickiewicz, Yoshikazu Kawai, Lauren Drage, Margarida C. Gomes, Frances Davison, Robert Pickard, Judith Hall, Serge Mostowy, Phillip D. Aldridge, Jeff Errington

**Affiliations:** 1 The Centre for Bacterial Cell Biology, The Institute for Cell and Molecular Biosciences, Medical School, Newcastle University, Baddiley-Clark Building, Newcastle upon Tyne, NE2 4AX UK; 2 Department of Immunology and Infection, London School of Hygiene and Tropical Medicine, Keppel Street, London, WC1E 7HT UK; 3 The Institute of Cellular Medicine, Newcastle University, 4th Floor, William Leech Building, Medical School, Framlington Place, Newcastle upon Tyne, NE2 4HH UK; 4 The Institute for Cell and Molecular Biosciences, Medical School, Newcastle University, Catherine Cookson Building, Framlington Place, Newcastle upon Tyne, NE2 4HH UK

**Keywords:** Antibacterial drug resistance, Cellular microbiology, Clinical microbiology, Pathogens

## Abstract

Recurrent urinary tract infection (rUTI) is a major medical problem, especially in the elderly and infirm, but the nature of the reservoir of organisms responsible for survival and recolonisation after antibiotic treatment in humans is unclear. Here, we demonstrate the presence of cell-wall deficient (L-form) bacteria in fresh urine from 29 out of 30 older patients with rUTI. In urine, *E. coli* strains from patient samples readily transition from the walled state to L-form during challenge with a cell wall targeting antibiotic. Following antibiotic withdrawal, they then efficiently transition back to the walled state. *E. coli* switches between walled and L-form states in a zebrafish larva infection model. The results suggest that L-form switching is a physiologically relevant phenomenon that may contribute to the recurrence of infection in older patients with rUTI, and potentially other infections.

## Abstract (web summary)

The reservoir for recurrent urinary tract infection in humans is unclear. Here, Mickiewicz et al. detect cell-wall deficient (L-form) *E. coli* in fresh urine from patients, and show that the isolated bacteria readily switch between walled and L-form states.

## Introduction

Urinary tract infection (UTI) is a major cause of human disease costing an estimated $1.6 billion per year in the United States^1^. Antibiotics are routinely used either to treat acute episodes or prevent recurrence; however, they occasionally fail to resolve the infection^2,3^. Recurence can be due to reinfection or persistence of bacteria. Bacterial survival during antibiotic treatment is generally thought to be facilitated by the formation of quiescent intracellular reservoirs of persister cells in the epithelium of the bladder^4,5^. Following antibiotic withdrawal, the bacteria can resume replication, causing full-blown infection^4,5^.

Bacterial cells are normally surrounded by a highly conserved structure called the cell wall. This layer, composed of peptidoglycan, defines their shape, facilitates regular division and protects from environmental factors, including changes in osmolarity^6^. Cell-wall components are recognised by the immune system and act as key indicators of infection^7^. The cell wall is also a target for immune effectors, such as lysozyme, as well as for some of our best and most commonly used antibiotics, particularly β-lactams, such as penicillins and cephalosporins^7,8^.

Several scientific reports from the 1960s to 1980s suggested that bacteria causing rUTI might survive treatment with cell-wall-specific antibiotics by adopting a cell-wall-deficient or L-form state^9–14^. However, these reports were mainly unconvincing, primarily due to the lack of molecular diagnostic tools^14^. Also, L-forms are almost completely overlooked in clinical settings because routine microbiological culture media are hypotonic and do not support L-form growth. We recently demonstrated that the L-form state can be induced by cells of the immune system in tissue culture, as well as in an animal model, rendering the bacteria resistant to cell-wall-targeting antibiotics^8^. In this report we show that L-forms are prevalent in older individuals suffering from rUTI. Furthermore, we demonstrate, in both urine and transparent zebrafish larvae, that *E. coli* strains isolated from these older patients can readily be induced to switch into the L-form state, to survive and divide, and then efficiently switch back into the walled state, potentially providing a route to the recurrence of infection.

## Results

### L-forms frequently occur in elderly rUTI patients

**Fig. 1:**
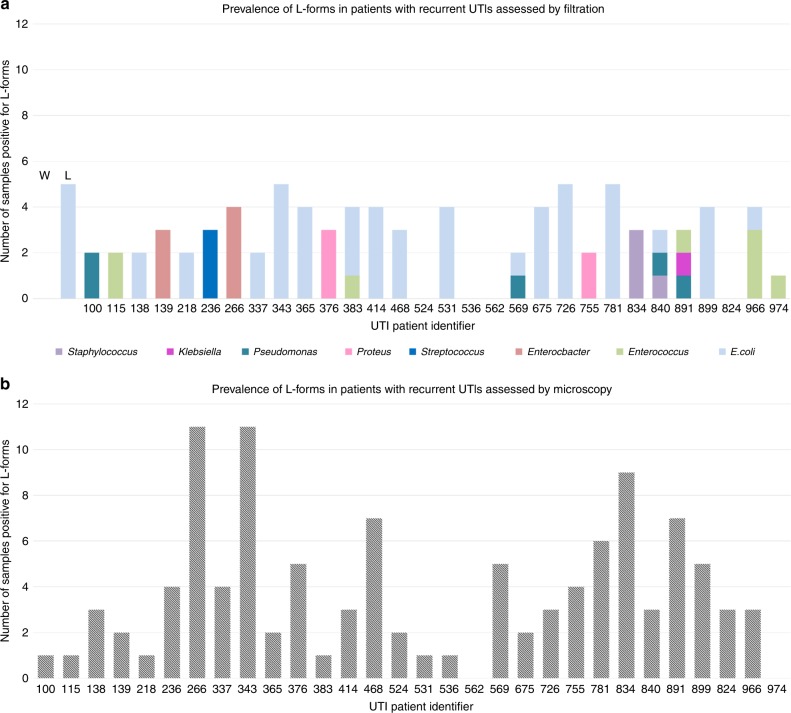
Prevalence of L-forms in older patients with recurrent UTIs. **a** Samples assessed by filtration. Each of the 30 recruited patients (*X* axis) donated a total of 12 samples over the period of 6 months (*Y* axis). Samples which contained filterable bacteria (some of which were also observed by microscopy), are symbolised by columns marked with solid colour (**a**). Various colours represent species of the filtered bacteria identified by 16S sequencing. W stands for a negative control in which 12 samples of laboratory BW25113 *E. coli* walled bacteria were passed through the 0.45 µm cut off filter onto osmoprotective media. L stands for a positive control where 12 samples of laboratory *E. coli* BW25113 L-form bacteria were filtered. **b** Samples assessed by phase contrast microscopy. Samples positive for L-form-like structures when assessed by microscopy are represented by grey, dashed columns

In a study described in detail elsewhere, 30 elderly patients suffering from rUTI donated samples of urine every 2 weeks over a period of 6 months, generating a bank of 360 samples^15^. Ability to pass through 0.45 μm cut off filters, which block the passage of walled forms of pathogens, has been used to detect L-forms in various settings^16–19^. A portion (2 ml) of each urine sample was filtered into semi-liquid osmoprotective media and incubated for up to 1 month. In many of the samples (Fig. 1a; Supplementary Fig. 1), growth detectable by eye appeared within 1–2 weeks, with some appearing after 3 days. Microscopically the bacteria resembled rods or cocci, which most likely originated from L-forms that passed through the filter, reverted to the walled state^19^ and resumed division by conventional binary fission (see also below). It is possible that immune factors or antibiotics which induced those L-forms in vivo, were sufficiently diluted or inactivated following filtration, which allowed bacteria to rebuild the wall. Given the slow emergence of growth it is improbable that this originated from passage of walled bacteria through damaged filters. Also, given the heavy bacterial load in most samples (typically around 10^3^–10^5^ viable bacteria per ml^15^), filter breakage would have led to much higher rates and levels of growths in the cultures than was seen. In control experiments cultures of walled *E. coli* did not detectably pass through the filters (which are generally used to sterilise solutions that cannot be autoclaved). Also, the recovery of *E. coli* L-forms (strain BW25113, induced under laboratory conditions) after filtration was inefficient (~41%) (Fig. 1a L), probably because most of the cells burst or were otherwise damaged by pressure and or torinal stress during filtration, so the number of positive samples was probably underestimated.

Sixty one percent of bacteria recovered after passage through the filter were identifiable as *E. coli* by 16S ribosomal sequencing; the remainder were mainly common UTI organisms, such as *Enterococcus* spp, *Klebsiella* spp, or *Proteus* spp (Supplementary Fig. 1B, Supplementary data 1)^20^. Multiple positive samples from the same patient usually contained bacteria of the same species (Fig. 1a).

We also examined all of the fresh urine samples directly by phase contrast microscopy. Although many classical rods and cocci were evident, especially in samples that did not coincide with antibiotic treatment of the patient, we also frequently detected polymorphic entities resembling L-forms, usually present side by side with walled bacteria. With one exception (patient UTI562), we found L-form-like structures in at least one of the 12 samples donated by each patient (Fig. 1b).

**Fig. 2:**
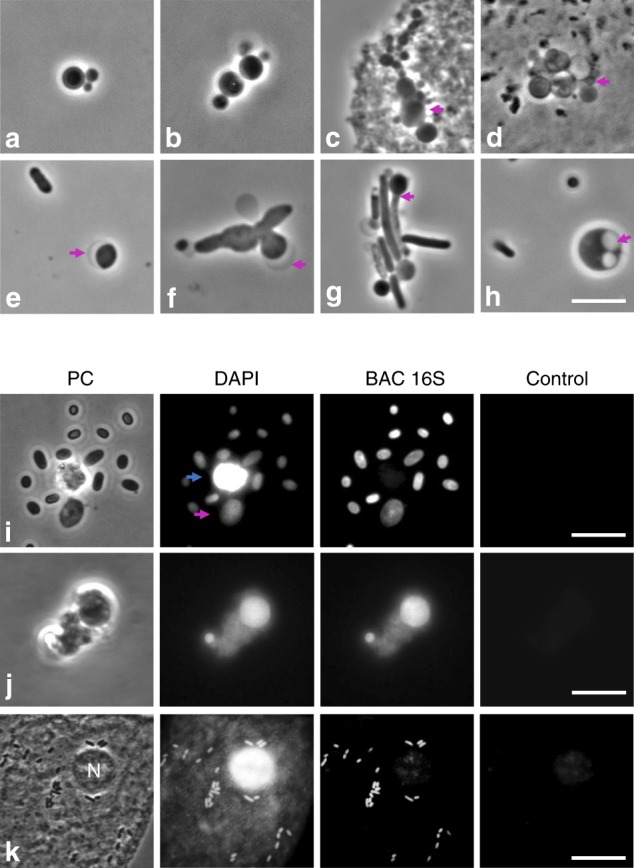
L-form-like structures observed in the urine of various patients. **a**–**h** Examples of fresh urine samples assessed by phase contrast microscopy. Bundels typical of dividing L-forms (**a**, **b**). Magenta arrows in (**c**) and (**d**) point to putative L-form bacteria associated with sluffed eukaryotic cells; in (**e**) and (**f**) they point at crescent shaped bulges, reminiscent of the outer membrane in L-forms of Gram-negative bacteria; in (**g**) a possible intermediate stage between walled and L-form is marked; and in (**h**), internal membrane vesicles are evident. **i**–**k** Examples of fixed samples assessed by fluorescence in situ hybridisation (FISH). Patient samples were fixed with paraformaldehyde and stained with DAPI and a fluorescently labelled DNA probe against bacterial 16S rRNA (BAC 16S). To show the specificity of DAPI and the probe staining an image was acquired in the red channel, which did not produce significant fluorescence (Control). **i** Two L-form-like structures were observed among several likely walled bacteria. One of the objects stained both with the bacterial probe and DAPI (magenta arrow) while the other one, only with DAPI (blue arrow). **j** Example of L-form-like structures of varied size that stained with the bacterial probe. **k** Example showing a eukaryotic cell in urine in the same field of view as numerous bacteria. The bacterial DNA and the nucleous of the eukaryotic cell (**n**) both stained with DAPI but the BAC 16S probe only associated with the bacterial cells, demonstrating the specificity of the probe. Scale bars = 5 µm, apart form panel (**k**), where the scale bar = 15 µm

Figure 2a–h shows representative examples of L-form-like structures observed in the urine samples. Spherical entities of various sizes, occasionally associated in bundles characteristic of dividing L-forms, were frequent, either suspended in urine (Fig. 2a, b) or associated with eukaryotic cells (Fig. 2c, d, magenta arrows). Occasionally, cells with phase pale crescent shaped bulges, which are typical of Gram-negative L-forms (probably due to local separation of inner and outer membranes) were observed (Fig. 2e, f, magenta arrows). What appeared to be intermediate stages between L-forms and walled bacteria were also frequently detected (Fig. 2f, g, magenta arrows). Sporadically, we observed spherical objects with internal membrane vesicles, which are characteristic of large L-forms (Fig. 2h, magenta arrow).

To confirm that the observed L-form-like objects were of bacterial rather than human origin (e.g. apoptotic bodies or various granules) we fixed 10 randomly selected samples of the urine and stained them with DAPI, which binds to DNA indiscriminately of origin, and with a fluorescently labelled oligonucleotide probe that hybridises with bacterial 16S rRNA transcripts (BAC 16S). Figure 2i–k contains typical examples of the results obtained. Figure 2i shows that polymorphic structures reminiscent of L-forms observed in the samples (magenta arrow) stained both with DAPI and the BAC 16S probe, just like rod-shaped (walled) bacteria. Occasionally we observed structures that stained only with DAPI indicative of eukaryote rather than bacterial origin (Fig. 2i, blue arrow). An example in Fig. 2k shows that the BAC 16S probe stained the bacteria but not human cell components. Crucially, in the FISH probed samples, virtually all of the objects (~99%) that looked like L-forms stained positively with the BAC 16S probe (Fig. 2i, j), confirming the bacterial origin of the observed polymorphic structures.

Overall, L-forms were detected by microscopy or filtration in 46% of collected samples (Fig. 1a, b). Even though accurate quantitation of the microscopic samples was difficult, we nonetheless readily detected L-form-like objects in samples derived from 20 µl of urine, and so we estimate that their frequency ranged from about 10^2^ to 10^4^/ml.

### L-forms provide a route for antibiotic evasion

**Fig. 3:**
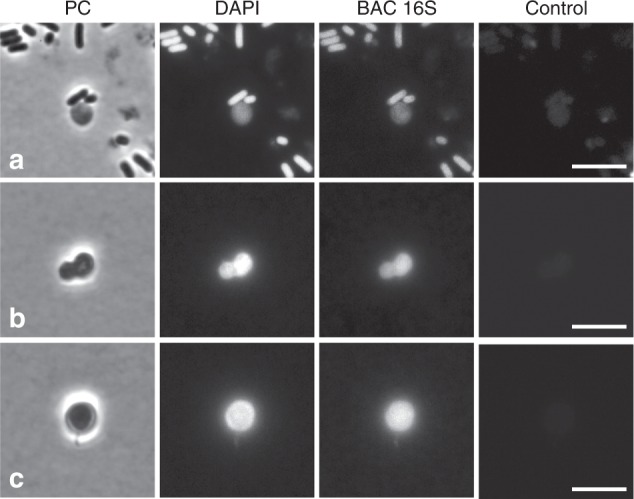
Examples of L-form-like structures observed in the patient UTI343 urine. **a**–**c** Example images taken before (**a**) and during (**b**, **c**) the treatment with phosphomycin, assessed by phase contrast and FISH. Scale bar = 5 µm

The fact that L-forms are completely resistant to antibiotics targeting the cell wall has been highlighted as contributing to the survival of bacteria during treatment^9–14^. We chose samples from patient UTI343 for further detailed analysis because the patient had been treated with phosphomycin (donation 6), which we have shown previously to actively promote L-form switching and proliferation^21,22^. A significant viable bacterial load (>1 × 10^5^) was detected by standard culture methods in all urine samples except from some in periods when the patient was being treated with antibiotics (Table 1). Multi-locus sequence typing (MLST) of cultured organisms revealed that the samples predominantly contained a common uropathogenic *E. coli* strain classified as type ST144^23^ (Supplementary data 1). Importantly, the same organisms were detected by conventional culture and after filtration, consistent with the idea that the organisms exist in both forms in the patient. The routine culture load was reduced 10-fold at first donation when the patient was treated with amoxicillin. In sample donations 5 and 11 when the patient was being treated with cephalexin, a different *E. coli* strain, less commonly implicated in UTI, referred to as ST782^24^ (Supplementary data 1), became apparent but ST144 was also detected. The ratio of ST144 to ST782 isolates detected in donations 5 and 11 was 1/5 and 1/2, respectively, based on 20 colonies screened. The viable bacterial load was reduced on those days to 300 and 5.6 × 10^4^/ml, respectively. A total of five samples contained bacteria that went through the filter, including on two occasions when the patient was being treated with cephalexin. No *E. coli* strains were detected by standard culture or filtration experiments in donation 6, when the patient was being treated with phosphomycin. However, L-forms were detected on that day by microscopy and unlike other samples (Fig. 3a), there were no rod-shaped bacteria among the potential L-forms (Figure 3b, c). The bacterial identity of these L-form-like objects was verified by FISH (Fig. 3). Notably, the urinary bacterial load returned to significance (>1 × 10^5^/ml) after the treatment was concluded and the strains associated with the relapse were ST144 and ST782.

**Table 1:** Recurrence of *E. coli* strains in patient UTI343 treated with phosphomycin (summary of results)

Donation	CFU/ml routine culture	L-forms observed	Treatment for UTI	Strains identified by routine culture	Strains identified following filtration
1	7 × 10^4^	YES	A	ST144	
2	1 × 10^5^	YES		ST144	ST144
3	1 × 10^5^	YES		ST144	ST144
4	1 × 10^5^	YES		ST144	
5	300	YES	C	ST144 ST782	ST144 ST782
6	CLEAR	YES	Pho		
7	1 × 10^5^	YES		ST144	
8	1 × 10^5^	YES		ST144	ST144
9	1 × 10^5^	YES		ST144	
10	1 × 10^5^	YES		ST144	
11	5 × 10^4^	YES	C	ST144 ST782	ST782
12	ND	ND	ND	ND	ND

First column indicates donation number. Second column contains the number of *E. coli* bacteria detected in each sample by a routine clinical culture method. Column three indicates whether structures reassembling L-forms were detectable by phase contrast microscopy in the urine samples immediately after they arrived at the lab. Column four shows whether the patient was treated with antibiotics for UTI: A-amoxicillin, C-cephalexin, Pho-phosphomycin. Column five shows which strains were identified by MLST in the samples from routine culture. Column six shows which strains were identified by MLST in the samples cultured following filtration

*ND* not done (sample not provided for this study)

### *E. coli* switches between L-form and walled states

**Fig. 4:**
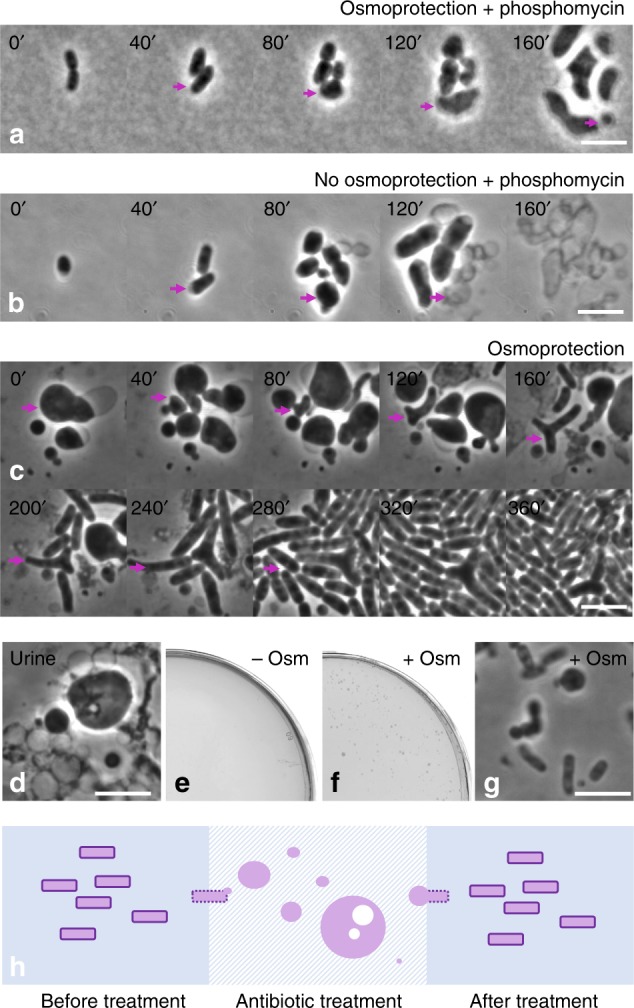
*E. coli* transition from rod to L-form and back on osmoprotective medium and in urine. Magenta arrows indicate individual bacteria, which were followed in time lapse and described in details in the main text. **a** Transition of strain ST782 from walled to L-form on osmoprotective medium (LM: L-form media) in the presence of 0.4 mg/ml phosphomycin. **b** Growth of strain ST782 in the presence of 0.4 mg/ml phosphomycin on non-osmoprotective medium (NA: nutrient agar). **c** ST782 transition from L-form to walled on osmoprotective medium (LM) after phosphomycin was removed. Time lapse over 5 h. Forty-minute increments are shown on the left-hand side of each image. **d**–**g** Survival of L-forms in urine and reversion to the walled state. **d** Phase contrast image of ST144 L-forms, which survived in urine overnight in the presence of phosphomycin. Viability of L-forms in urine was demonstrated by plating on NA (**e**) and LM (**f**). Growth was detectable only on osmoprotective medium (**f**) after 24 h incubation at 37 °C. **g** Microscopic appearance of bacteria detected in (**f**). **h** Model showing L-form switching as a mechanism for the recurrence of bacterial infections. Bacteria causing UTI are treated with cell-wall-targeting antibiotics. This leads to elimination of the cell wall and emergence of L-forms, which are not detectable by standard clinical culture methods. Following antibiotic treatment the bacteria can regenerate the wall and potentially cause recurrence of a full-blown infection. The bacterial cell wall is indicated with dark purple lines. Panel (**h**) was created by Katarzyna Mickiewicz. Scale bars = 5 µm

Transition from the walled state to the L-form has been shown for a wide range of bacteria but usually L-form growth is much slower than that of walled bacteria and requires osmoprotective conditions^14^. We have recently shown that immune effectors such as lysozyme can induce an L-form switch^8^. Furthermore, certain antibiotics that interfere specifically with cell-wall precursor synthesis, such as phosphomycin or D-cycloserine, can also induce L-forms^21,22^. Given that L-form-like bacteria were found in many of the patient samples, it was important to determine whether these bacteria are capable of an efficient L-form switch and growth. To test this, an *E. coli* ST782 isolate from above was subjected to time-lapse microscopy in the presence of phosphomycin, on media with (Fig. 4a, Supplementary Movie 1) or without (Fig. 4b, Supplementary Movie 2) osmoprotection. In the absence of osmoprotection the cells underwent several conventional divisions. They then started to bulge, and all of the cells had lysed within 2.5 h. In contrast, on osmoprotective media, after bulging, the cells underwent a series of typically erratic L-form division events and continued to grow in this state. The L-form transition was completed within about 3 h of incubation.

To provide a source of recurrent infection L-forms would need to be capable of returning back to the walled state in the absence of antibiotic. To test this, we placed L-forms on osmoprotective medium, without phosphomycin. Typical time-lapse imaging results are shown in Fig. 4c and Supplementary Movie 3. The L-forms initially went through several rounds of typical irregular divisions. After 40 min, cells with an elongated shape and of approximately normal diameter for walled cells began to appear. Some of the L-forms contained appendages protruding in different directions (Fig. 4c magenta arrow). The tubular cells elongated and eventually began to divide by the classical binary fission characteristic of walled cells. The initial L-form to rod transition required only about 1.5 h of growth following withdrawal of phosphomycin and after 5 h virtually all bacteria in the field of view had returned to the walled state. Similar results were obtained for other strains, e.g. ST144 (Supplementary Movies 4, 5 and 6).

### Urine supports L-form switching

L-form switching would only be relevant to the clinical situation if the L-forms were viable in urine or tissues. One of the concerns is whether those environments could provide sufficient osmoprotection for L-form survival. The osmolality of urine of a healthy individual oscillates between about 400 and 800 mOsm/kg^25,26^, similar to that of the osmoprotective media we use to grow L-forms (500 mOsm/l). To test whether patient isolates have the potential to survive as L-forms in urine, we placed walled ST144 bacteria on an agarose pad made with the filtered urine of a healthy individual supplemented with phosphomycin and observed the cells by time-lapse microscopy. Supplementary Movie 7 shows that the bacteria were able to switch into the L-form state, and that they could proliferate, albeit less efficiently than in optimised L-form growth media (most likely due to lower nutrient levels in urine). To test cell viability and their potential to regenerate the wall, we placed ST144 L-forms in liquid filtered urine supplemented with phosphomycin and incubated overnight. The presence of intact L-forms the next day was confirmed by phase contrast microscopy (Fig. 4d). The L-forms were plated on media with and without osmoprotection and incubated at 37 °C for 24 h. A lawn of small variable colonies emerged only on the osmoprotective plates (Fig. 4e, f). Microscopic examination revealed predominantly walled bacteria, though L-forms and intermediate types were also observed (Fig. 4g). These bacteria could now grow well on non-osmoprotective medium showing that they had switched back to the walled state. These results demonstrate that L-forms of bacteria isolated from a patient with rUTI can survive in urine in the presence of phosphomycin and are able to regenerate their cell wall following antibiotic withdrawal (Fig. 4h). Rojas et al.^27^ recently showed that the Gram-negative outer membrane contributes substantially to the mechanical stability of the cell, perhaps explaining how well the L-forms appear to survive in urine.

### L-forms can regenerate the cell wall in vivo

**Fig. 5:**
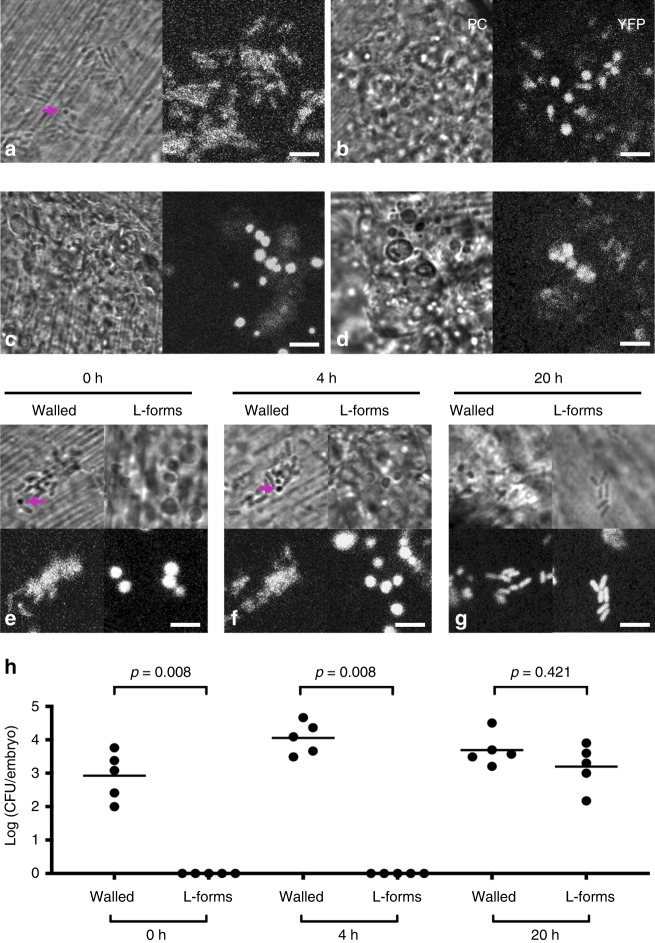
L-form switching in the zebrafish embryo. **a**, **b** Walled *E. coli* ST144-YFP was injected into the tail fin of zebrafish larvae in the presence (**b**) or absence (**a**) of 0.4 mg/ml phosfomycin and visualised by phase contrast (PC) or fluorescence microscopy (YFP). The magenta arrow in (**a**) points to a bacterium that appears to have adopted a round shape in the absence of antibiotic. **c**, **d** ST144-YFP *E. coli* L-forms induced in vitro with 0.4 mg/ml phosfomycin were injected into the tail fins of zebrafish larvae in the presence of 0.4 mg/ml phosfomycin and visualised by microscopy immediately post-injection (**c**), or following an overnight incubation (**d**). **e**–**g** ST144-YFP *E. coli* walled or in vitro induced L-form bacteria were injected in the absence of the antibiotic and visualised 0 h (**e**), 4 h (**f**) or 20 h (**g**) post infection. **h** The bacteria were enumerated by homogenising fish embyos and plating out on NA 0, 4 or 20 h post infection. Each circle represents recovered bacteria from an individual larva. Representative data from two independent experiments (one with two and one with three larvae). Mean (horizontal bars) is shown. The *p* values were determined by non-parametric Mann–Whitney test. Significance was defined as *p* < 0.05. Scale bars = 5 µm. Source data for Fig. 5h are provided as Source Data file

Transparent zebrafish (*Danio rerio*) larvae allow direct visualisation of bacteria at the single cell level in a living higher organism. To look directly for L-form switching in vivo, a derivative of a ST144 *E. coli* isolate expressing cytoplasmic YFP was injected into the tail fin of zebrafish larvae at 3 days post fertilisation (dpf) in the absence (Fig. 5a) or presence (Fig. 5b) of phosphomycin, and visualised using phase contrast (PC) or fluorescence (YFP) microscopy. (Note that the striations observed in the phase contrast images are cartilage structures of the larval tail fin.) In the absence of antibiotic, most of the walled bacterial cells retained their rod shape (Fig. 5a), although some occasionally became rounded (Fig. 5a, magenta arrow). This morphological switching, which could indicate progression to L-form or dying cells, could have occurred spontaneously or have been due to the activity of host phagocytic cells, such as neutrophils and macrophages, that damage the bacterial cell wall. However, in the presence of phosphomycin, switching to a characteristic L-form morphology occurred rapidly (~30 min post infection) and in virtually all injected larvae (Fig. 5b). Supplementary Fig. 2A and Supplementary Movie 8 show the progression of a rod-shaped bacterium (magenta arrow) to a round shape over 40 min during the course of imaging. Although some bacteria progressed to L-form faster than others, these experiments showed that switching to the L-form can occur rapidly in vivo in the presence of phosphomycin.

To test for L-form survival in vivo, ST144-YFP *E. coli* L-forms induced in vitro with phosphomycin were injected into larvae in the presence of the antibiotic, and visualised as described above. In this case, L-forms were clearly visible immediately following the injection as rounded entities of various sizes (Fig. 5c). Moreover, many L-forms remained easily detectable following overnight incubation (Fig. 5d), showing that long term survival of cell-wall-deficient bacteria is supported in the complex and innate-immune-proficient environment of zebrafish larvae.

To further test whether L-form bacteria could survive in that state in vivo, but also switch back into walled forms, phosphomycin induced ST144-YFP L-forms were washed to remove the antibiotic and then injected into larvae in the absence of antibiotic; walled bacteria were injected into another set of larvae as controls. The injection sites were visualised by fluorescence microscopy to verify the presence of bacteria 0, 4 and 20 h post infection (Fig. 5e–g). In parallel, bacteria were recovered from larvae on NA medium (to detect walled cells) (Fig. 5h)). For larvae injected with walled bacteria, microscopy confirmed the presence of rod-shaped bacteria at all time points, and again rounded bacteria were occasionally observed (magenta arrows) (Fig. 5e–g, walled). In agreement with this, walled bacteria were consistently recovered at all time points tested (Fig. 5h, walled). In the case of larvae injected with L-forms, no CFU were detected at 0 and 4 h post infection (Fig. 5h, 0 h and 4 h, L-forms), even though L-forms were observed at those times by microscopy (Fig. 5e, f, L-forms). However, bacterial colonies started to emerge 20 h post infection (Fig. 5h, 20 h, L-forms) at which point rod-shaped (i.e. walled) bacteria were also detected by microscopy (Fig. 5g, L-forms). To test for switching of L-forms in vivo, larvae injected with L-forms were visualised using time-lapse microscopy following overnight incubation. Supplementary Fig. 2B and Supplementary Movie 9 show a rounded cell resembling an L-form followed over 30 min, during which the bacterium changed shape from round into the elongated shape characteristic of walled cells. Supplementary Fig. 2C and Supplementary Movie 10 show another rod-shaped bacterium followed over 80 min in a separate region of the same larva undergoing two rounds of division by binary fission, characteristic of walled bacteria. Thus, L-forms can not only survive for many hours in vivo, but also regenerate their wall and resume normal growth and division in the zebrafish infection model.

## Discussion

This pilot scale study of clinical samples has demonstrated that L-forms are frequently present in the urine of a cohort of older patients with rUTI. Although, many of these patients were treated with cell-wall-active antibiotics this cannot explain their presence in samples taken during periods with no antibiotic treatment. We have recently shown that immune effectors such as lysozyme can actually promote the L-form switch, at least for several Gram-positive bacteria^8^. The occasional detection of rounded cells in the zebrafish experiments would be consistent with innate-immune generation of L-forms. Although the zebrafish model is useful for visualising the walled to L-form transition in vivo, mammalian models will be needed to characterise the possible role of the L-form in recurrent infection going forward. Ultimately, larger scale trials focused specifically on culture negative patients treated with cell-wall-targeting antibiotics will be required to directly establish the extent to which L-form switching contributes to antibiotic evasion.

Aside from antibiotic evasion (discussed below) L-form bacteria may also benefit from being less susceptible to innate-immune killing than walled cells. Although they may still be susceptible to other arms of the immune system, for example antimicrobial peptides or immune response stimulated by LPS, they presumably do not trigger innate-immune receptors specific for cell-wall components^7^. Overall, it seems likely that the immune stimulation by L-forms is less than that exerted by walled bacteria. It is conceivable that L-forms can survive particularly well in immunocompromised or older patients.

One of the most significant implications of this study is that, along other mechanisms used by bacteria to recur, L-forms could provide a source of bacterial survivors during treatment with cell-wall-specific antibiotics, independent of the need to acquire specific resistance genes (Fig. 4h). Instead, the antibiotic target (the cell wall) is temporally removed and its requirement for survival bypassed. Unlike previously described dormant persister cells^4,5^, L-forms can continue to proliferate during what can be long (typically 5–14 days) periods of antibiotic treatment. Most importantly, we directly show that L-form bacteria induced by the presence of antibiotic in urine or zebrafish larvae are able to revert to a walled state once the antibiotic is removed. Beyond rUTI it now seems plausible that L-form switching might be an underappreciated mechanism of antibiotic tolerance by bacteria in other recurrent or chronic infections^9–14^. Finally, our results suggest that effective clearance of some recurrent infections might require the combination of cell-wall-targeting antibiotics with other classes of antibiotics, such as those targeting the bacterial membrane.

## Methods

### Patients

Urine samples were obtained from a pilot study of 30 geriatric (age 65 or over) individuals with a history of recurrent UTI, run under ethical approval of the Newcastle Research Ethics Committee (reference REC-14-NE-0026)^15^. Patients were recruited with written informed consent through UTI clinics led by the Urology Department at Freeman Hospital, Newcastle upon Tyne, UK. Volunteers were from either weekly UTI clinics led by the Urology Department at the Freeman Hospital, Newcastle-upon-Tyne or the organisation VoiceNorth (www.voicenorth.org). Inclusion criteria stated that patients were community dwelling and needed to have had two or more symptomatic UTIs as noted in their clinical records. Exclusion criteria specified evidence of complicated UTI such as catheter use.

The samples were donated every 2 weeks for a period of 6 months and screened in an unbiased manner with respect to clinical data. The study was designed such that patients should provide both symptomatic and non-symptomatic samples, the latter serving as internal controls.

### Bacterial strains and growth conditions

CPS Elite diagnostic plates (BioMerieux) or L-form media (LM) supplemented with 0.2% agarose were used for initial culture of bacteria from urine samples. LM media contained 1x Brian Heart Infusion (BHI) supplemented with 0.5 M sucrose and 8 mM MgSO_4_. For growth of isolated ST144 and ST782 *E. coli* strains, ST144-YFP and laboratory *E. coli* strain BW25113 nutrient broth (NB, Oxoid) or nutrient agar (NA, Oxoid) was used as non-osmoprotective medium and liquid L-form medium (LM) or supplemented with agarose at 0.2 or 1% as indicated, were used for osmoprotection. Fresh urine of a healthy individual passed through a 0.2 µm cut off filter (supplemented with 0.2% agarose for time-lapse microscopy) was used for urine survival assays. Phosphomycin was added at 400 µg/ml where indicated. The cultures were incubated at 37 or 30 °C as indicated. *yfp* plasmid PMCL200^28^ was introduced to electrocompetent ST144 cells by electroporation at 2.5 µF, 200 Ω, 2.5 V in a Gene Pulser Xcell electroporation unit (Bio-Rad) to create strain ST144-YFP.

### Urine processing

Urine processing and scoring for the presence of L-forms was blinded. Fresh urine samples obtained from patients were streaked using 10 µm loops on CPS Elite plates and incubated overnight at 37 °C. Aliquots of 2 ml were filtered through 0.45 µm cut off filters onto 5 ml of LM medium in 30 ml universal tubes, supplemented with 0.2% agarose and incubated at 30 °C for up to 1 month. Samples cultured in semi-liquid medium were re-streaked on NA plates and single colonies were picked for 16S rRNA sequencing. For patient UTI343, 20 colonies were subjected to MLST sequencing prior to and after filtration. Samples of fresh urine (500 µl) were concentrated 20× by centrifugation at 8000 × *g* for 2 min and a 1 µl volume from each sample was examined immediately by phase contrast microscopy. Samples of 250 µl urine were fixed with 4% paraformaldehyde, followed by overnight incubation at 4 °C. Paraformaldehyde was removed and the samples were stored in 50% ethanol/50% PBS at −20 °C.

### Zebrafish injections

All zebrafish experiments were performed using wild-type AB zebrafish. ﻿Eggs were obtained from naturally spawning zebrafish and embryos were reared in Petri dishes, at 28.5 °C in embryo medium (0.5X E2) supplemented with 0.3 µg/ml methylene blue^29^. For injections and live imaging, 3 days post fertilisation (dpf) larvae were anaesthetised with 200 µg/ml tricaine (Sigma-Aldrich) in embryo medium. During in vivo microscopy, larvae were maintained in a 33 °C atmosphere, for optimal conditions for bacterial replication. Bacterial strain ST144-YFP was cultured overnight in liquid NB, supplemented with 30 µg/ml chloramphenicol, and sub-cultured to exponential phase (OD_A600_ = 0.6). For L-forms injections, sub-cultures in NB were supplemented with 0.4 mg/ml phosphomycin. To prepare the inoculum for injection, the suspension was centrifuged for 4 min at 4000 × *g*, washed and resuspended in PBS to 10,000 CFU/nl. Larvae were injected with 1–2 nl of bacterial suspension in the tail fin. For induction of L-forms in vivo, 0.4 mg/ml phosphomycin was injected intravenously (1 nl) and added to the embryo medium containing anaesthetic. Larvae were sacrificed in tricaine and mechanically disrupted in LM liquid media at the indicated time points. For recovery experiment (Fig. 5c), homogenates were serially diluted and plated onto NA, supplemented with 30 µg/ml chloramphenicol, to recover rod bacteria. Each circle represents recovered bacteria from an individual larva (two independent experiments, one with two and one with three larvae).

### Ethics statements

Animal experiments were performed according to the Animals (Scientific Procedures) Act 1986 and approved by the Home Office (Project licences: PPL P84A89400 and P4E664E3C). All experiments were conducted up to 5 days post fertilisation.

### Sequencing

Genomic DNA of bacteria isolated following filtration was extracted using a DNeasy Blood and Tissue kit (Qiagen) and subjected to sequencing with the following pairs of primers:

16S rRNA (performed on all samples):^30^

E334F forward CCAGACTCCTACGGGAGGCAGC

E1115R reverse AGGGTTGCGCTCGTTG

MLST (performed on *E.coli* strains isolated from patient UTI343):^31^

adenylate kinase:

adk_forward ATTCTGCTTGGCGCTCCGGG

adk_reverse CCGTCAACTTTCGCGTATTT

fumarate hydratase:

fumC_forward TCACAGGTCGCCAGCGCTTC

fumC_reverse GTACGCAGCGAAAAAGATTC

DNA gyrase:

gyrB_forward TCGGCGACACGGATGACGGC

gyrB_reverse ATCAGGCCTTCACGCGCATC

isocitrate/isopropylmalate dehydrogenase:

icd_forward ATGGAAAGTAAAGTAGTTGTTCCGGCACA

icd_reverse GGACGCAGCAGGATCTGTT

malate dehydrogenase:

mdh_forward ATGAAAGTCGCAGTCCTCGGCGCTGCTGGCGG

mdh_reverse TTAACGAACTCCTGCCCCAGAGCGATATCTTTCTT

adenylosuccinate dehydrogenase:

purA_forward CGCGCTGATGAAAGAGATGA

purA_reverse CATACGGTAAGCCACGCAGA

ATP/GTP binding motif:

recA_forward CGCATTCGCTTTACCCTGACC

recA_reverse TCGTCGAAATCTACGGACCGGA

### Microscopy imaging

For snapshot live-cell imaging, urine or cells were mounted on microscope slides directly or on 1% agarose in water, NB or LM. For time-lapse imaging, cells were placed in 25 µl Gene Frames (Thermo Fisher) on 0.2 or 0.5% agarose in NB, LM or urine. The slides were placed on the microscope stage at 30 °C. Images were acquired with a Rolera EM-C2 (Q-imaging) camera attached to a Nikon TiE microscope, and analysed using Metamorph (Molecular Devices). For zebrafish imaging, embryos were embedded in 1% low-melting-point agarose in 35 mm diameter glass-bottom MatTek dishes. In vivo time lapses were acquired using Zeiss LSM 880 and ×63 oil immersion objective. Pictures and videos were prepared for publication using ImageJ. Representative images were included in the figures.

### Fluorescence in situ hybridisation (FISH)

Fixed samples from the frozen at −20 °C were thawed and serially dehydrated with 60, 80 and 100% ethanol followed by hybridisation with a FISH probe specific for bacterial 16S rRNA: BAC 16S GCTGCCTCCCGTAGGAGT (referred to as EUB338 in ref. ^32^) at 46 °C for 4 h in a hybridisation buffer containing 1 M NaCl, 20 mM Tris-HCl, pH 7.5, 0.1% SDS and 20% formamide. Following hybridisation, the probes were placed in wash buffer containing 1 M NaCl, 20 mM Tris-HCl, pH 7.5, 0.1% SDS and incubated for 15 min at 48 °C. The wash was repeated 2×. After washing, samples were resuspended in 10 µl water, placed on a 1% agarose water pad containing 1 µl/ml DAPI and visualised by fluorescence microscopy.

### Urine survival assay

ST144 bacteria were streaked on LM plates containing phosphomycin and incubated overnight at 30 °C. The resulting L-forms were placed the next day in fresh filtered urine containing phosphomycin and incubated overnight at 37 °C. The following day, integrity of the L-forms was assessed by phase contrast microscopy and the culture OD_600_ was adjusted to 0.5. Hundred microliters of samples were plated on NA or LM plates and incubated for 24 h at 37 °C. Emerging colonies were examined by phase-contrast microscopy.

### Reporting summary

Further information on research design is available in the Nature Research Reporting Summary linked to this article.

## Supplementary information

Supplementary information

Description of Additional Supplementary Files

Supplementary Data 1

Supplementary Movie 1

Supplementary Movie 2

Supplementary Movie 3

Supplementary Movie 4

Supplementary Movie 5

Supplementary Movie 6

Supplementary Movie 8

Supplementary Movie 7

Supplementary Movie 9

Supplementary Movie 10

Reporting Summary

Source data

## Data Availability

16S rRNA and MLST data are available at GenBank with accession numbers provided in Supplementary Data 1. The source data underlying Fig. 5H are provided as a Source Data file.
