# Author Correction: Possible role of L-form switching in recurrent urinary tract infection

**DOI:** 10.1038/s41467-019-13256-5

**Published:** 2019-11-20

**Authors:** Katarzyna M. Mickiewicz, Yoshikazu Kawai, Lauren Drage, Margarida C. Gomes, Frances Davison, Robert Pickard, Judith Hall, Serge Mostowy, Phillip D. Aldridge, Jeff Errington

**Affiliations:** 1 The Centre for Bacterial Cell Biology, The Institute for Cell and Molecular Biosciences, Medical School, Newcastle University, Baddiley-Clark Building, Newcastle upon Tyne, NE2 4AX UK; 2 Department of Immunology and Infection, London School of Hygiene and Tropical Medicine, Keppel Street, London, WC1E 7HT UK; 3 The Institute of Cellular Medicine, Newcastle University, 4th Floor, William Leech Building, Medical School, Framlington Place, Newcastle upon Tyne, NE2 4HH UK; 4 The Institute for Cell and Molecular Biosciences, Medical School, Newcastle University, Catherine Cookson Building, Framlington Place, Newcastle upon Tyne, NE2 4HH UK

**Keywords:** Antibacterial drug resistance, Cellular microbiology, Clinical microbiology, Pathogens

**Correction to**: *Nature Communications* 10.1038/s41467-019-12359-3, published online 26 September 2019.

The original version of this Article contained an error in Fig. 2k, in which the control panel was inadvertently duplicated from that of Fig. 2j. The correct panel for Fig. 2k has been included in both the PDF and HTML versions of the Article.

