# Supplementary material for: Possible role of L-form switching in recurrent urinary tract infection
Source: Nat Commun. 2019 Sep 26;10:4379. doi: 10.1038/s41467-019-12359-3 (PMC6763468; doi:10.1038/s41467-019-12359-3)
Supplement: Supplementary file 1 — Supplementary information [file 41467_2019_12359_MOESM1_ESM.pdf]

Supplementary Information

"Possible role of L-form switching in recurrent urinary tract infection"

Mickiewicz et al.

Supplementary Figure 1

A

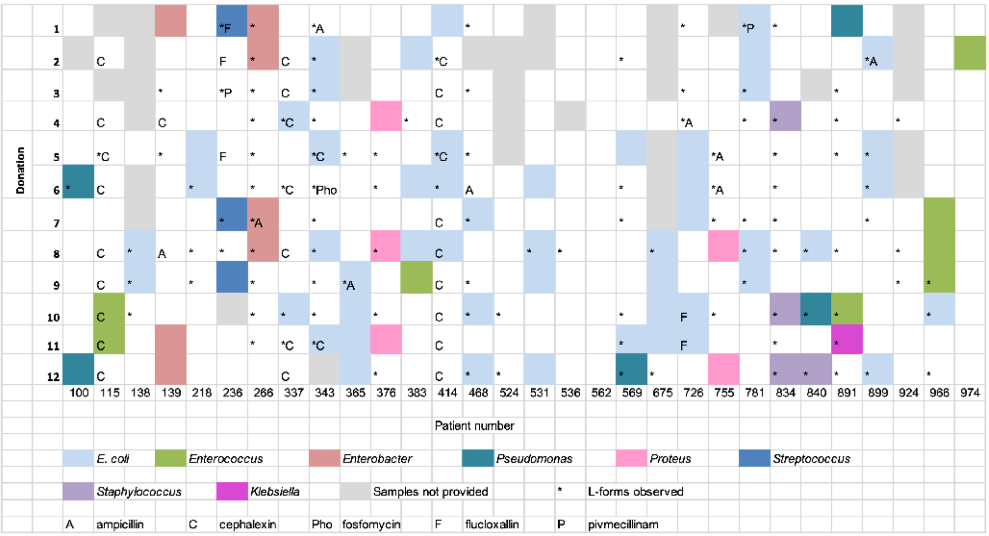

B

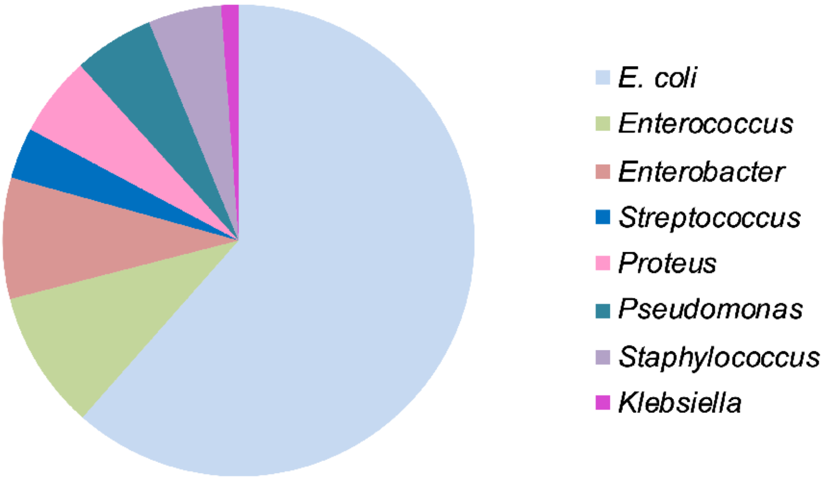

Supplementary figure 1. Compilation of data for all of the patient samples. A) On the x axis is displayed patient number and on the y axis donation number. Asterixes indicate whether L-forms were observed in the sample by microscopy. Samples that went through the filter are colour coded and various colours indicate which bacterial species was identified following 16S sequencing. Letters indicate various cell wall targeting antibiotics that patients were treated with during donation. Grey boxes indicate samples that were not provided for this study. B) Distribution of bacterial species that were recovered after filtration.

Supplementary Figure 2

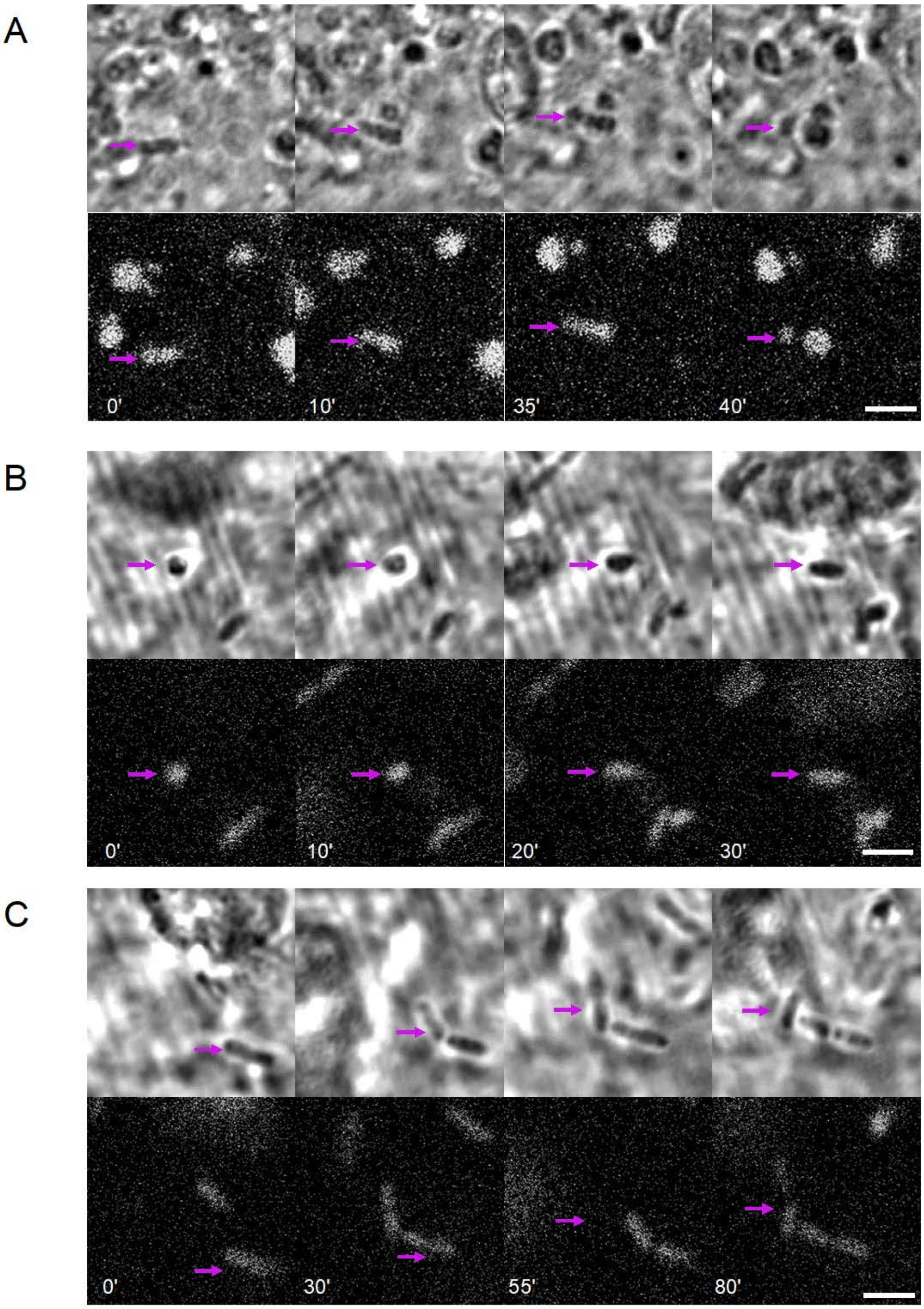

Supplementary figure 2. L-form switching in zebrafish larvae. A) *E. coli* ST144-YFP was visualised by phase contrast and fluorescence microscopy during a period of 40min in zebrafish larvae following injection into the tail fin in the presence of phosphomycin. The magenta arrow points at a cell that underwent a change in shape from rod to spherical, which is associated with the switch from walled form to L-form. B) The transition of an *E. coli* ST144-YFP L-form to the walled form over 30 min following overnight incubation in a zebrafish larva. C) *E. coli* ST144-YFP division by binary fission in a zebrafish larva.
