# Supplementary material for: Possible role of L-form switching in recurrent urinary tract infection
Source: Nat Commun. 2019 Sep 26;10:4379. doi: 10.1038/s41467-019-12359-3 (PMC6763468; doi:10.1038/s41467-019-12359-3)
Supplement: Supplementary file 2 — Description of Additional Supplementary Files [file 41467_2019_12359_MOESM2_ESM.pdf]

Description of Additional Supplementary Files

File Name: Supplementary Data 1
Description: GenBank accession numbers for 16s and MLST sequencing data.

File Name: Supplementary Movie 1
Description: *E. coli* ST782 switching from the rod to L-form state on osmoprotective medium in the presence of phosphomycin.

File Name: Supplementary Movie 2
Description: *E. coli* ST782 switching from the rod to L-form state on non-osmoprotective medium in the presence of phosphomycin.

File Name: Supplementary Movie 3
Description: *E. coli* ST782 switching from the L-form to rod state on osmoprotective medium.

File Name: Supplementary Movie 4
Description: *E. coli* ST144 switching from the rod to L-form state on osmoprotective medium in the presence of phosphomycin.

File Name: Supplementary Movie 5
Description: *E. coli* ST144 switching from the rod to L-form state on non-osmoprotective medium in the presence of phosphomycin.

File Name: Supplementary Movie 6
Description: *E. coli* ST144 switching from the L-form to rod state on osmoprotective medium.

File Name: Supplementary Movie 7
Description: *E. coli* ST144 switching from the rod to L-form state in urine in the presence of phosphomycin.

File Name: Supplementary Movie 8
Description: *E. coli* ST144-YFP switching from the rod to L-form state in a zebrafish larva.

File Name: Supplementary Movie 9
Description: *E.coli* ST144-YFP switching from the L-form to rod state in a zebrafish larva.

File Name: Supplementary Movie 10
Description: *E.coli* ST144-YFP division by binary fission in a zebrafish larva.
